# Late presentation of chronic HBV and HCV patients seeking first time specialist care in Spain: a 2-year registry review

**DOI:** 10.1038/s41598-021-01885-0

**Published:** 2021-12-17

**Authors:** Camila A. Picchio, Sabela Lens, Manuel Hernandez-Guerra, Juan Arenas, Raúl J. Andrade, Javier Crespo, Javier García-Samaniego, Manuel Romero-Gómez, Juan Turnes, José Luis Calleja, Miguel Ángel Simón, Trenton M. White, Mar Riveiro-Barciela, Anna Pocurull, Dalia Morales-Arraez, Alexandra Gómez, Maria Buti, Jeffrey V. Lazarus

**Affiliations:** 1 Barcelona Institute for Global Health (ISGlobal), Hospital Clínic, University of Barcelona, Barcelona, Spain; 2 Liver Unit, Hospital Clinic, Universidad de Barcelona, Barcelona, Spain; 3 IDIBAPS, University of Barcelona, Barcelona, Spain; 4 CIBER Hepatic and Digestive Diseases (CIBERehd), Instituto Carlos III, Madrid, Spain; 5 Department of Gastroenterology, University Hospital of the Canary Islands, San Cristóbal de La Laguna, Spain; 6 Biodonostia, Liver Diseases Research Group, Osakidetza Basque Health Service, Donostia University Hospital, San Sebastián, Spain; 7 Unidad de Gestión Clínica de Enfermedades Digestivas, Instituto de Investigación Biomédica de Málaga-IBIMA, Hospital Universitario Virgen de la Victoria, Universidad de Málaga, Málaga, Spain; 8 Gastroenterology and Hepatology Unit, Hospital Universitario Marqués de Valdecilla, IDIVAL, University of Cantabria, Santander, Spain; 9 Liver Unit, Department of Gastroenterology, Hospital Universitario La Paz, IdiPAZ, Madrid, Spain; 10 UCM Digestive Diseases, Hospital Universitario Virgen del Rocío, Institute of Biomedicine of Seville, Universidad de Sevilla, Seville, Spain; 11 Complejo Hospitalario Universitario de Pontevedra, Instituto de Investigación Sanitaria Galicia Sur (IISGS), Pontevedra, Spain; 12 Gastroenterology and Hepatology Department, Hospital Universitario Puerta del Hierro de, Universidad Autónoma de Madrid, Madrid, Spain; 13 Department of Digestive Diseases, Hospital Clínico de Zaragoza, Zaragoza, Spain; 14 Instituto de Investigación Sanitario Aragón (IIS Aragón), Zaragoza, Spain; 15 Liver Unit, Hospital Universitario Vall d’Hebron, Barcelona, Spain

**Keywords:** Hepatology, Health services, Hepatitis B, Hepatitis C

## Abstract

Chronic viral hepatitis infection affects an estimated 325 million people globally. People who initiate treatment after significant disease progression face increased risk of severe liver complications and death. Data are scarce on the characteristics and risk factors of people who present late to care in Spain and globally. Data were collected from January 2018 to December 2019 to report late presentation (LP) to specialist care at 11 large university hospitals in Spain to assess related risk factors using a multivariable logistic regression model. 2290 (CHB = 505, CHC = 1785) patients were analysed, with 581 (25.2%) presenting late. Hepatitis C patients more frequently reported LP compared to hepatitis B patients (28.1% vs 15.0%; p < 0.001). Older age (p < 0.001), being male (p < 0.001), being Spanish-born (p < 0.001), and having an unknown origin of referral (p = 0.08) were associated with a higher likelihood of LP. Advanced liver disease was identified in 533 (23%) patients and late-stage liver disease in 124 (5.4%). LP, including with irreversible liver damage, to viral hepatitis specialist care is frequent in Spain, despite being a country with unrestricted treatment access. Initiatives to reduce LP should specifically target men, older individuals, foreign-born populations for CHB, and Spanish nationals for CHC.

## Introduction

Viral hepatitis currently affects an estimated 325 million people globally^1^. In 2016, the World Health Organization (WHO) adopted the “*Global Health Sector Strategy on Viral Hepatitis, 2016–2021*”, which sets out to eliminate viral hepatitis as a public health threat by 2030 by reaching targets that principally aim to reduce new hepatitis infections by 90% and deaths by 65%^2^. Recent data in the WHO European region report that an estimated 15 million people live with chronic hepatitis B virus (HBV)^3^ and an estimated 14 million people with hepatitis C virus (HCV), equivalent to one in 50 people affected^4^. Diagnosis can be challenging as chronic HBV and HCV infections may remain clinically silent for decades with symptoms only occurring once the disease has significantly progressed.

HBV and HCV infected individuals are at an increased risk of developing liver cirrhosis, decompensated liver disease, hepatocellular carcinoma (HCC), and liver-related death^5, 6^. The risk of disease progression is related to several factors with viral replication being an important factor in fibrosis progression and thus, in the risk of developing HCC. Therefore, antiviral therapy that can suppress viral replication in the case of hepatitis B and direct-acting antivirals (DAAs) against HCV^7^, can modify the natural course of the disease and reduce or prevent the development of disease progression, particularly if therapy is initiated in early stages of the disease. In the case of chronic hepatitis C (CHC) infection, although antiviral therapy results in sustained virologic response (SVR) in ≥ 95% of cases, for patients that present late to care, there is still a risk of HCC for those that have developed advanced fibrosis or cirrhosis^8, 9^. In Spain, access to HCV DAA therapy has been unrestricted since mid-2017, yet only half of the patients with HCV-RNA know their status and are receiving therapy^10^. Viral elimination through completion of DAA treatment therapy can reduce all-cause mortality in HCV patients^11^. Similarly, timely diagnosis and treatment initiation of patients with HBV is required to prevent patients from progressing to severe adverse clinical outcomes, which is estimated to be as high as 15–40%^12^.

For virtually all transmissible infectious diseases, late presentation (LP) to care has both individual and population health implications. Screening to identify these individuals before significant liver damage occurs has reduced HBV and HCV incidence^13^ and prevalence and has been shown to maintain or improve quality of life^14^. Thus, to optimise the benefits of available treatment regimens and work towards the WHO elimination goal, patients need to be diagnosed earlier. In a 2017 consensus paper^15^, LP was defined as: (1) presentation with advanced liver disease (ALD) in untreated patients with chronic hepatitis B and C showing significant fibrosis (F ≥ 3); or (2) presentation with late-stage liver disease (LSLD) in untreated patients with chronic hepatitis B or C and including at least one symptom of decompensated cirrhosis (i.e., jaundice, hepatic encephalopathy, clinically detectable ascites, variceal bleeding) and/or HCC in patients with no previous antiviral treatment.

There are few studies evaluating late presentation in general, and population level studies are largely unexplored. The aim of this study is to report the prevalence of LP of chronic infection with HBV and HCV at first visits with specialists prescribing therapy and managing HBV and HCV in Spain, which has unrestricted treatment access, and describe the risk factors associated with HBV and HCV late presentation to care.

## Methods

This is a retrospective (Jan 2018–Feb 2019) and prospective (Mar–Dec 2019) cohort study (data collection was first retrospective and subsequently prospective) of adult patients (18 years or older) with chronic HBV or HCV infection presenting for consultation with a liver specialist in 11 Spanish university hospitals in eight of Spain’s 17 autonomous communities from 1 January 2018 to 31 December 2019. Chronic HCV infection was defined by the presence of HCV-RNA for > 6 months and chronic HBV infection by HBsAg detection for > 6 months^16, 17^. Patients with acute hepatitis as well as those with acute liver failure were excluded. Those presenting for care with a liver specialist (hepatology department, infectious diseases department, internal medicine department, or “other”) were referred by a primary care office or other specialist. In Spain, only specialists are permitted to treat and manage chronic viral hepatitis patients and, therefore, referrals from other departments are needed before being seen by a specialist. Data from each patient’s visits with specialists were collected in a non-identifiable Excel collection template through review of medical records in each hospital’s medical record system by authorised personnel. Standard demographic variables (gender, nationality, date of birth), epidemiological (mode of transmission, year of diagnosis, vaccination status), laboratory parameters (alanine aminotransferase [ALT], aspartate aminotransferase [AST], platelet count), viral hepatitis B and C serologies and viral load (HBsAg, HBeAg, HBV-DNA, HCV-RNA, HCV genotype and subtype), fibrosis stage, and clinical outcome variables, such as decompensated cirrhosis and hepatocellular carcinoma (HCC), were collected. Variables on the type of centre and information on referral of patients were also collected. Late presentation was defined based on the consensus definition proposed by Mauss et al.^15^ (Box 1).

**Box 1:** Consensus definition of late presentation with chronic viral hepatitis for medical care.

Presentation with advanced liver disease (ALD) in untreated patients with chronic hepatitis B and C	A patient with chronic HBV or HCV and significant fibrosis assessed by one of the following: serologic fibrosis score ≥ 3 (assessed by APRI score > 1.5, FIB-4 > 3.25, Fibrotest > 0.59 or alternatively transient elastography (FibroScan) > 9.5 kPa or liver biopsy (≥ METAVIR stage F3) in patients with no previous antiviral treatment
Presentation with late-stage liver disease (LSLD) in untreated patients with chronic hepatitis B and C	A patient with a fibrosis score > F3 and presence of at least one symptom of decompensated cirrhosis (jaundice, hepatic encephalopathy, clinically detectable ascites, variceal bleeding) and/or hepatocellular carcinoma in patients with no previous antiviral treatment

*Source *Mauss et al.^15^.

*APRI* aspartate aminotransferase to platelet ratio, *F3* stage 3 fibrosis, *FIB-4* Fibrosis 4, *HBV* hepatitis B virus, *HCV* hepatitis C virus, *METAVIR* Meta-analysis of Histological Data in Viral Hepatitis.

### Variables

The date of birth of patients were collected and later birth year was extracted in order to calculate age at time of presentation to care. Country of origin was re-categorised into “Spanish-born” and “Foreign-born” and consisted of responses as “Spain” or “España” versus all other possible nationalities. HBV-DNA and HCV-RNA were recorded as UI/mL, ALT and AST values were recorded as UI/L, and platelet counts were standardised and recorded as × 10^3^/µL. The upper limit of normality (ULN) for ALT and AST values was set at 40 UI/mL. HBeAg and HBsAg were collected dichotomously as “positive” or “negative” for HBV patients. In the case of HCV-related variables, genotype letter and number were collected. Fibrosis stage was reported as F0-F4 and later re-categorised to create the variable “advanced liver disease”, which included all patients with fibrosis ≥ F3 and an aminotransferase platelet ratio (APRI) score > 1.5 based on the initial (first or second) patient visits with the liver specialist. Patients who did not have a reported fibrosis stage had their APRI index score calculated using reported AST and platelet count values using the standard formula [(AST/upper limit of the normal AST range) × 100/platelet count]. The variable LSLD included any patient with a reported liver complication (jaundice, hepatic encephalopathy, ascites, variceal bleeding) defining decompensated cirrhosis and/or HCC in addition to a fibrosis score > F3. If fibrosis stage or HCC or liver complications were not available, the patient entry was excluded from analysis. Mode of transmission was collected categorically and included: sexual, same sex; sexual, hetero; IV drug use; blood transfusion; other; unknown. In the instance of HBV-related variables, being correctly vaccinated against HBV was collected as “yes,” “no,” and “unknown”. Year of diagnosis and was collected as a continuous variable in years. If an HCV patient had a reported year of diagnosis prior to 1989 (year of the discovery of the virus), this was considered a diagnosis of “non-A non-B” hepatitis. Origin of patient referral was also collected categorically (primary care; other specialty in the same centre; gastroenterology or hepatology specialist from another centre; other specialist from another centre; other; unknown). Blank answers were treated as missing values. Any anomalies in databases were verified with the co-author from each corresponding centre to be verified and modified if needed. Data that did not meet inclusion criteria were removed from the database and excluded from analysis; under the age of 18 (n = 12); HBsAg negative (n = 4); ALT > 600 UI/L (n = 31); no data on liver fibrosis, HCC, or liver complications to calculate LP (n = 11).

### Statistical analyses

The prevalence of LP was calculated for the whole sample and stratified according to the following categories: chronic HBV or HCV infection, participant centre, sex, country of origin (Spanish-born vs foreign-born), mode of transmission, and origin of referral. Patients who presented late to care were compared to patients without late presentation. Binary logistic regression tested the odds ratios (OR) between late presentation and three variables of interest (sex, age, and country of origin). The level of significance was set to < 0.05, and data were analysed using Stata, version 16.

### Ethics approval and consent to participate

This study received ethical clearance in 2019 from the Ethical Committee of the Hospital Clínic, Barcelona, Spain (n. HCB/2019/0111), and was performed in accordance with relevant guidelines and regulations. No personally identifiable information was collected from study participants.

## Results

2290 patients were included in this study (HBV = 505, HCV = 1785). The mean age of the population was 53.8 years (SD: 14.4) and men represented 62.6% (n = 1434) of the population (Table 1). The majority (77.9%; 1783) of patients were Spanish-born and the majority of patients with viral hepatitis infection had an “unknown” (59.6%; 1366) mode of transmission, followed by injecting drug use (21.4%; 490). Most patients were referred to the liver specialist for the first time from primary care (44.4%; 1016), followed by an “other” referral (17.3%; 396). 8.8% (n = 203) of patients did not have a reported origin of referral. The mean year of diagnosis for 2199 patients with available data was 2011 (SD: 9.2) with a range of diagnosis year from 1966 to 2019. 54.4% (n = 1245) of the data were collected from a review of 2018 patient history and the remaining from 2019 (n = 1045). There were no changes in LP prevalence between the two years of data collection (25.9% vs 25.5%, respectively).

**Table 1:** Baseline characteristics of all patients included in the 11 Spanish centres, 2018/19.

	OVERALL (N = 2290) N = 2290	HBV (N = 505) N (%)	HCV (N = 1785) N (%)
**Age**, mean (SD)	53.8 (14.4)	46.2 (15.6)	55.9 (13.3)
**Sex**			
Male	1434 (62.6)	294 (58.2)	1140 (63.9)
Female	854 (37.3)	211 (41.8)	643 (36.0)
**Country of origin**	N = 2274		
Spanish-born	1783 (77.9)	224 (44.4)	1559 (87.3)
Foreign-born	491 (21.4)	275 (54.4)	2176 (12.1)
Missing values	16 (0.7)	6 (1.2)	10 (0.6)
**Mode of transmission**			
Sexual, same sex	23 (1.0)	9 (1.8)	14 (0.8)
Sexual, hetero	38 (1.7)	11 (2.2)	27 (1.5)
Injecting drug use	490 (21.4)	5 (0.9)	485 (27.2)
Blood transfusion	185 (8.1)	15 (2.9)	170 (9.5)
Other	149 (6.5)	78 (15.4)	71 (4.0)
Unknown	1366 (59.6)	385 (76.2)	981 (54.9)
Missing values	39 (1.7)	2 (0.4)	37 (2.1)
Year of diagnosis (mean, SD)	2011 (9.4)	2013 (8.9)	2010 (9.2)
**Origin of referral**			
Primary care	1016 (44.4)	276 (54.6)	740 (41.5)
Other specialty in the same centre	319 (13.9)	114 (22.6)	205 (11.5)
Gastrohep specialist from another centre	228 (9.9)	30 (5.9)	198 (11.1)
Other specialist from another centre	79 (3.4)	13 (2.6)	66 (3.7)
Other	396 (17.3)	11 (2.2)	385 (21.6)
Unknown	203 (8.8)	47 (9.3)	156 (8.7)
Missing values	49 (2.1)	14 (2.8)	35 (1.9)

*HBV* hepatitis B virus, *HCV* hepatitis C virus.

Late presentation was detected in 25.7% (n = 588) of all patients. ALD was reported in 552 (24.1%) patients and LSLD was reported in 5.3% (122) of all patients (Table 2). Overall, 15.7% (359) presented with stage F4 fibrosis and 7.4% (170) had stage F3 fibrosis at first consultation determined by Fb > 9.5 at most centres (1166; 50.9%) (Supplementary Table 1). An additional 23 (1%) who did not have fibrosis stage reported and had AST and platelet count values available had an APRI score greater than 1.5, indicating ALD. Mean ALT was 67.6 UI/L and AST was 57.6 UI/L. Seventy-five (3.3%) patients reported at least one liver complication defining decompensated cirrhosis. The most common reported liver complication was clinical ascites (17.9%; 35), followed by jaundice (12.3%; 24). An additional 113 “other” non-decompensated cirrhosis defining liver complications were reported.

**Table 2:** Description of HBV and HCV LP patients (N = 2290), categorised by ALD/LSLD in 11 Spanish hospitals.

	Overall	HBV (N = 505)	HCV (N = 1785)
	n (%)	n (%)	n (%)
**Late presentation to care**^**a**^			
Yes	588 (25.7)	78 (15.4)	510 (28.6)
Advanced liver disease	552 (24.1)	72 (14.3)	480 (26.9)
Missing values	10 (0.4)	2 (0.4)	8 (0.5)
**Fibrosis stage**			
F3	170 (7.4)	17 (3.4)	153 (8.6)
F4	359 (15.7)	48 (9.5)	311 (17.4)
APRI > 1.5	23 (1.0)	7 (1.4)	16 (0.9)
Late-stage liver disease	122 (5.3)	28 (5.5)	94 (5.2)
Hepatocellular carcinoma	55 (2.4)	10 (2.0)	45 (2.5)
Missing values	78 (3.4)	0 (0)	78 (4.3)
**Decompensated cirrhosis defining liver complications**^**b**^			
Yes	75 (3.3)	19 (3.8)	56 (3.1)
Jaundice	21 (0.9)	9 (1.8)	12 (0.7)
Hepatic encephalopathy	8 (0.3)	0 (0)	8 (0.4)
Ascites	37 (1.6)	11 (2.1)	26 (1.4)
Variceal bleeding	18 (0.8)	3 (0)	15 (0.8)
Missing values	214 (9.3)	133 (26.3)	186 (10.4)

*ALD* advanced liver disease, *APRI* aspartate aminotransferase to platelet ratio, *F3* stage 3 fibrosis, *F4* stage 4 fibrosis, *HBV* hepatitis B virus, *HCV* hepatitis C virus, *LP* late presentation, *LSLD* late-stage liver disease.

^a^Late presentation to care includes those with ALD and LSLD, of which, some patients may classify in both categories (i.e. a patient may have fibrosis F4 and a liver complication).

^b^Number of patients for each type of liver complication sum to more than the “YES” total because some patients reported more than one liver complication.

### Chronic HBV and HCV

Patients with chronic HCV infection were more likely to present late to care compared to those with chronic HBV (28.6% vs 15.4%; p < 0.001). ALD was reported more frequently in HCV patients compared to HBV patients (26.9% vs 14.3%; p < 0.001) and LSLD was described in 5.5% (n = 28) and 5.2% (n = 94) of HBV and HCV patients, respectively (p = 0.806). HCV patients had a higher mean ALT at baseline compared to HBV patients: 57 UI/L [95% CI 44.8–70.9] and 70 UI/L [95% CI 67.3–73.7]; p = 0.0069) and AST at baseline (59.9 UI/L [95% CI 57.2–62.6] and 49 UI/L [95% CI 40.2–59.5]; p = 0.0065). HBV patients presenting late to care had a significantly higher proportion of ALT and AST values above the upper limit of normality (≥ 40) in comparison to those not presenting late (ALT: 60.8% v. 26.9%; p < 0.001, AST: 60.8% v. 21.3%; p < 0.001). This same trend was similar among HCV patients (ALT: 78.2% v. 59.9%; p < 0.001, AST: 78.4% v. 50.1%; p < 0.001).

### Hepatocellular carcinoma (HCC)

55 (2.4%) patients presented with HCC at first consultation (Table 3). The majority of HCC patients were Spanish born and male. Those with HCC were significantly older than those without HCC (p < 0.001) and 81.8% of those with HCC at first consultation (n = 55) were chronic HCV patients (n = 45) (p < 0.001). Those with HCV infection and HCC on average were older than those with HBV infection and HCC (64.8 years vs. 56 years).

**Table 3:** Description of patients with and without HCC who presented late to care. Spain, 2018/2019.

	Patients with HCC (n = 55)	Patients without HCC (n = 2173)
**Age**, mean (SD)	63.2 (12.6)	53.4 (14.2)
**Sex**		
Male	42 (2.9)	1352 (94.3)
Female	13 (1.5)	803 (94.0)
**Country of origin**		
Spanish-born	50 (2.8)	1683 (93.7)
Foreign-born	5 (1.0)	474 (96.1)

Missing values not reported.

*HCC* hepatocellular carcinoma.

### Country of origin

**Map 1:**
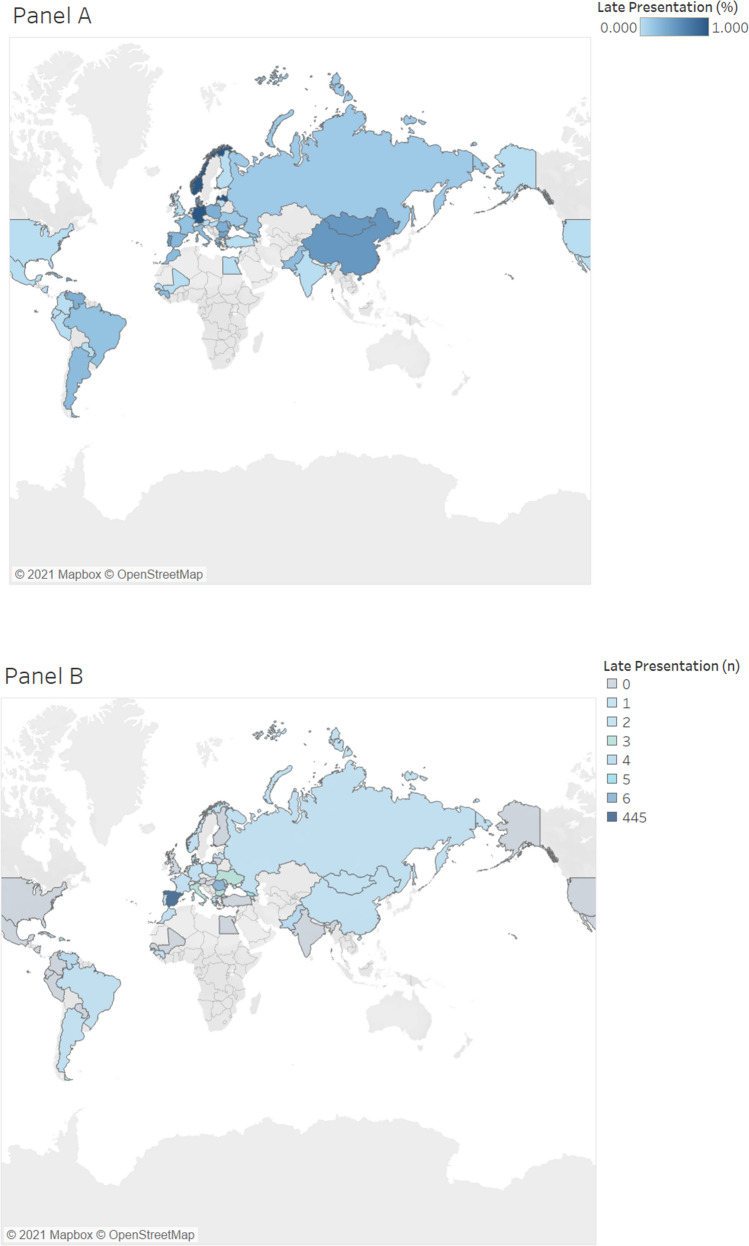
Percent of HCV LP patients by country of origin (**A**) and number of HCV LP patients (N = 510) from each corresponding country (**B**). *HCV* hepatitis C virus, *LP* late presentation. Map was created using Tableau version 2021.1.0 (www.tableau.com).

**Map 2:**
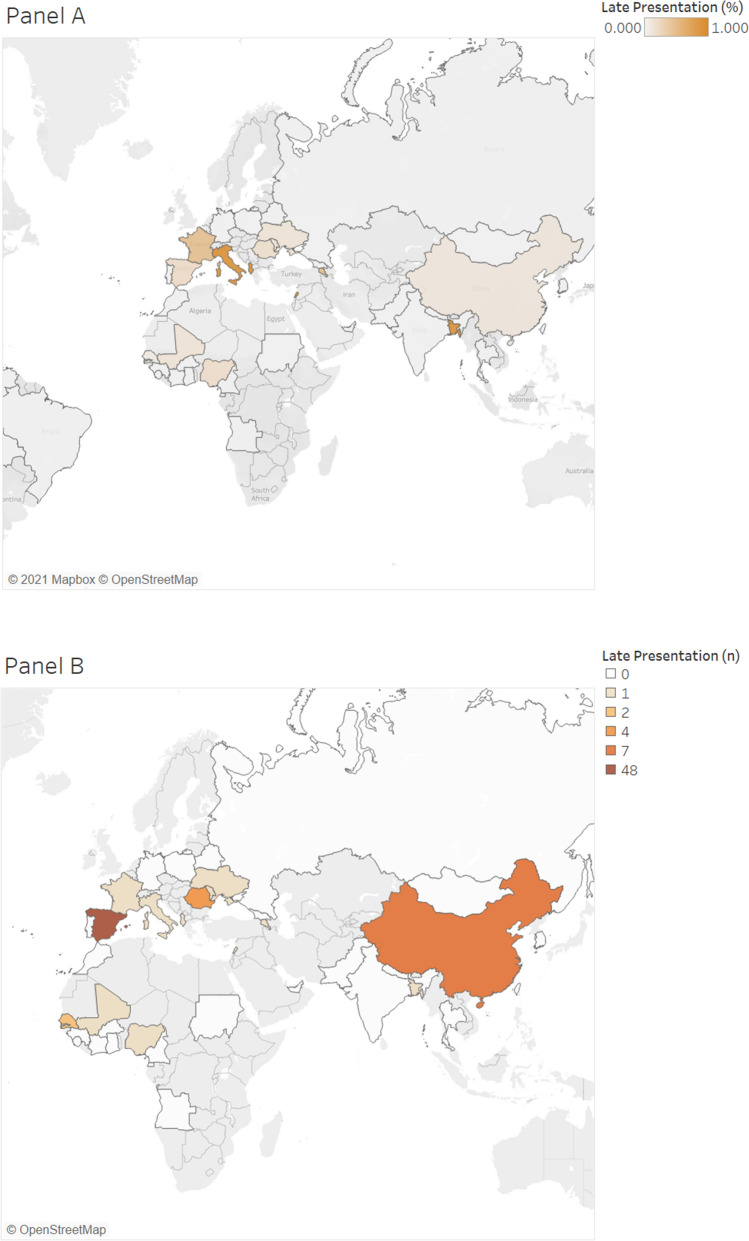
Percent of HBV LP patients by country of origin (**A**) and number of HBV LP patients (N = 78) from each corresponding country (**B**). *HBV* hepatitis B virus, *LP* late presentation. Map was created using Tableau version 2021.1.0 (www.tableau.com).

Of those that presented late to care, the majority (85.7%; 504) were Spanish-born individuals and foreign-born individuals made up 13.2% (n = 78) of those presenting late to care (p < 0.001). HCV patients were primarily Spanish-born individuals (87.3%; 1559) (Map 1) whereas about half of all HBV patients were foreign-born individuals (54.5%; 275) (Map 2). 34.6% (n = 27) of HBV patients who presented late to care were foreign-born (p = 0.001) compared to 10.0% (n = 51) of HCV patients (p = 0.079).

After Spain (1783), patients were primarily from China (66), Romania (39), Georgia (37), Pakistan (39), and Senegal (29). Patients from these mentioned countries all presented late to care (Maps 1 and 2). Chinese and Senegalese patients primarily had chronic HBV, representing 97% (64) and 96.5% (28) of their infections, respectively. In comparison, patients from Georgia primarily had chronic HCV (83.8%; 31). Romanian patients represented both chronic HBV (53.8%; 21) and HCV (46.2%; 18) infections. Similarly, patients from Pakistan also represented both chronic HBV (41.4%; 12) and HCV (58.6%; 17) infections.

### Age

Overall, those who presented late to care were older (59 years vs 51 years; p < 0.001) than those who did not present late. This trend did not change for patients with ALD (59 years vs 52 years; p < 0.001) or LSLD (59 years vs 53 years; p < 0.001). HCV patients were significantly older compared to HBV patients (56 years vs 46 years; p < 0.001).

### Sex

Men were more likely to present late to care compared to women (30.3% vs17.8%; p < 0.001). This trend did not change for patients with ALD where 28.7% (n = 412) of men and 16.3% (139) of women had ALD (p < 0.001). The proportion of patients who presented with LSLD was double in men (6.4%; 92) compared to women (3.5%; 30) (p = 0.011).

### Origin of referral

Patients who were referred from primary care made up 39.3% (n = 231) of patients who presented late to care (Table 4).

**Table 4:** Referral origin of LP patients, compared to those who did not present late (Spain, 2018/2019).

Origin of referral	Late presentation, n (%)	No late presentation, n (%)
Primary care	231 (22.7)	785 (77.3)
Other specialty in the same centre	90 (28.2)	229 (71.8)
GI specialist from another centre	67 (29.4)	161 (70.6)
Other specialist from another centre	24 (30.4)	55 (69.6)
Other	101 (25.5)	295 (75.5)
Unknown	64 (31.5)	139 (68.5)
Missing values	11 (22.4)	38 (77.6)
Total	588 (100)	1702 (100)

*LP* late presentation.

### Participating centres

Eleven tertiary hospitals, representing six out of the 17 (35%) autonomous communities in Spain, provided data. Late presentation to care ranged from 4.6% to 34% across the centres and autonomous communities (Table 5). HCV patients presented late to care more frequently than HBV patients across all centres.

**Table 5:** Proportion of HBV and HCV LP patients categorised by 11 participating Spanish centres, 2018/2019.

Hospital (city)	HBV patients n (%)*	% HBV patients with LP n (%)	HCV patients n (%)*	% HCV patients with LP n (%)	Total patients (n)	Overall LP n (%)
Hospital 1 (Tenerife)	36 (12.2)	6 (16.7)	260 (87.9)	68 (26.1)	296	74 (12.6)
Hospital 2 (Santander)	25 (18.4)	4 (16.0)	111 (81.6)	40 (36.0)	136	44 (7.6)
Hospital 3 (Madrid)	N/A	N/A	143 (100)	45 (31.5)	143	45 (7.6)
Hospital 4 (San Sebastián)	27 (10.6)	8 (29.6)	226 (89.3)	69 (30.5)	253	77 (13.1)
Hospital 5 (Barcelona)	125 (31.0)	16 (12.8)	278 (69.0)	39 (14.0)	403	55 (9.3)
Hospital 6 (Madrid)	21 (17.8)	4 (19.0)	97 (82.2)	29 (29.9)	118	33 (5.6)
Hospital 7 (Pontevedra)	N/A	N/A	136 (100)	26 (19.1)	136	26 (4.4)
Hospital 8 (Sevilla)	48 (35.8)	13 (27.1)	86 (64.2)	33 (38.4)	137	46 (7.8)
Hospital 9 (Barcelona)	163 (38.6)	10 (6.2)	258 (61.4)	90 (34.8)	420	100 (17.0)
Hospital 10 (Málaga)	47 (30.7)	16 (34.0)	106 (69.3)	45 (42.4)	153	61 (10.4)
Hospital 11 (Zaragoza)	14 (14.3)	1 (7.1)	84 (54.7)	26 (30.9)	98	27 (4.6)

*N/A* not available—did not provide HBV data.

*HBV* hepatitis B virus, *HCV* hepatitis C virus, *LP* late presentation.

*(%) reported in relation to the total of all patients in the hospital.

Bivariate logistic regression analyses with late presentation as the outcome (Table 6) were conducted for sex, age, origin of referral, and country of origin (Spanish-born vs. foreign-born). Being female was associated with a 50% decreased odds of LP and being Spanish born was associated with double the odds of presenting late. Each additional year of age corresponded to 0.3% greater odds of LP. Compared to primary care as the origin of referral, each of the other origins of referral showed greater odds of late presentation and in particular, having an unknown origin of referral had the greatest odds of LP.

**Table 6:** Odds ratios and their 95% confidence intervals univariate logistic regressions for late presentation.

Variable	Odds ratio (OR)	95% Confidence interval	p-value
Age	1.03	[1.03–1.04]	< 0.001
**Sex**			
Male (reference group)	–	–	< 0.001
Female	0.49	[0.40–0.61]	
**Country of origin**			
Foreign-born (reference group)	–	–	–
Spanish born	2.08	[1.60–2.71]	< 0.001
**Origin of referral**			
Primary care (reference group)	–	–	–
Other specialty in the same centre	1.33	[1.00–1.77]	0.046
GI specialist from another centre	1.41	[1.02–1.94]	0.034
Other specialist from another centre	1.48	[0.89–2.44]	0.124
Other	1.16	[0.88–1.52]	0.271
Unknown	1.56	[1.12–2.17]	0.008

*GI* gastrointestinal.

## Discussion

Late presentation to hepatitis specialist care (liver units or gastroenterologist specialists) continues to be a challenge in Spain, with nearly one-fourth of the patients in this study of 11 large university hospitals presenting with advanced liver disease or late-stage liver disease at first consultation with a liver specialist. Patients with chronic hepatitis C infection presented late to care more frequently than those with hepatitis B and were predominantly Spanish-born individuals.

Late presentation to care results in more advanced disease progression at baseline, making treatment (for both HBV and HCV) and subsequent cure (in the case of HCV) more challenging. In Spain, cirrhosis and other chronic liver diseases due to HCV and HBV accounted for 1598 deaths (0.38% of total) and 618 (0.15%) deaths in 2017, respectively. Liver cancer accounted for 2860 (0.69%) deaths due to HCV and 409 (0.1%) deaths due to HBV in the same year^17^. Health systems must ensure that those who are in need of treatment are screened, diagnosed, and linked to specialist care before their liver disease progresses to cause substantial liver damage^18^. In the instance of human immunodeficiency virus (HIV), a definition of late presentation at the time of diagnosis has aided in surveillance and the identification of risk factors for presenting late with HIV^19^ in addition to monitoring trends over time^20^.

Delayed linkage to care or loss to follow-up after referral to specialised care poses a significant barrier to timely treatment initiation^21^ and poses additional risk for ALD and LSLD. A delayed referral for specialised treatment from primary care, where viral hepatitis diagnoses are often made, extends the amount of time individuals live undertreated or untreated with either or both diseases, highlighting deficiencies within the health system. Not only should screening for viral hepatitis be strengthened at the primary care level, but the referral pathway needs to be improved. In our study, we identified patients being referred from other specialists in the same hospital or even other gastroenterology and hepatology services from other hospitals. The former suggests that referral processes in the hospitals need to be strengthened, which is a healthcare failure. The latter possibly highlights that some patients may have difficult-to-treat hepatic conditions, rather than them presenting late to viral hepatitis care. This nuance should be considered by healthcare providers at the point of care engagement, when, despite presenting with ALD or LSLD, patients may not have initially presented late to care. Strong care pathways (e.g. referral processes) are a particular challenge during the ongoing COVID-19 pandemic as primary care centres in Spain are overburdened with COVID-19 cases, and may have less capacity and resources to address other morbidities, including viral hepatitis. In our study, the mean year of patients’ diagnosis was 2011; 7–8 years prior to reaching specialist care with a gastroenterologist or hepatologist despite possibly having been in hospital care for comorbidities. Patients who present with hepatocellular carcinoma at first consultation with a specialist initiate treatment once the impact of prolonged and untreated viral hepatitis infection has already resulted in irreversible liver damage. Deaths due to cirrhosis constituted 2.4% of total deaths globally in 2017^22^. In a single-centre study conducted in Denmark, 5.3% (28/527) of patients presenting late (n = 169) had LSLD at first consultation^23^. Our study reported a similar prevalence of LSLD (5.8%), of which 55 patients presented with HCC at first consultation between the years 2018 and 2019. Similarly, in a study using newly-diagnosed hepatitis B related HCC patients (n = 1276) in Korea, Sinn et al. reported that in 2013, 23.1% of patients with newly diagnosed HCC cases had no prior clinic visits before their HCC diagnosis^24^. In contrast, in 2003, this prevalence was 50.9%, highlighting the impact of effective targeted screening in Korea. However, any proportion of LP, and specifically of HCC at first consultation, highlights that timely diagnosis and referral needs to be optimized and targeted as a public health challenge.

In the study by Sinn et al*.*^24^*,* authors reported that a lower income was associated with a higher risk of LP. Our study did not collect income level variables; however, it would be interesting to further explore potential barriers to accessing specialised care in Spain, which could include socioeconomic barriers. Healthcare is free and universal for all legal residents of Spain in all autonomous communities of the country so cost of care would not be anticipated to be a main factor contributing to LP to care. However, HBV patients were predominantly foreign-born individuals, particularly from sub-Saharan African and Asian countries. Migrants who have irregular legal status in the country would not be entitled to access the public healthcare system, which could be a risk factor contributing to the 15.0% of LP HBV cases in our study. Furthermore, 35% of all HBV patients who presented late to care were not Spanish natives, highlighting the importance of early detection of HBV among migrant populations residing in Spain. In addition, HBV vaccination coverage of foreign-born individuals was not well reported across all centres, with nearly 50% reporting incorrect vaccination among patients and an additional 40.8% in missing values (data not reported in results). Incomplete or incorrect vaccination of HBV among patients arriving to Spain from endemic countries should suggest an urgency for timely HBV screening. Proper vaccination coverage reporting will consolidate current monitoring and surveillance methods and may detect gaps in testing and referral services.

In contrast, HCV patients were predominantly Spanish natives (87.3%) and had a history of past or current injecting drug use (27.1%). ALD was reported in 26.9% of HCV patients, similar to those described in another cohort study (GECCO cohort, Germany) which reported an ALD prevalence of 32.5%^25^. However, 210/863 of the patients included in the GECCO cohort were HIV/HCV co-infected. HIV patients are increasingly aware of their status and are regularly in care, which would suggest that screening for HCV would be done in a timely manner, reducing risk for LP. Integrated care is a reported best practice for increasing screening, management, and treatment monitoring for HCV care^26^ and in Spain, elimination of HCV among those who are HIV/HCV is feasible^27^, as is the case in other settings^28^. Of those who had a documented mode of transmission in our study, injecting drug use was the most common route of HCV infection. Targeted interventions for this key population should be implemented if lacking in particular regions and scaled up widely to reach the WHO 2030 elimination targets.

Notably, however, our study also had a large proportion of unknown modes of transmission, both for HBV and HCV patients. This poses a particular challenge when designing targeted interventions for key populations, as described in the Spanish Ministry of Health screening guidelines^29^. Age was identified as a risk factor for LP and the presence of hepatocellular carcinoma in our study, which can serve as a basis for increased screening among older age groups, including multiple generations, similar to what is recommended in the United States^30^.

Current patient-centred models of care exist across Spain to increase testing and linkage to care for people who inject drugs. These models of care have high cure rates and should be expanded, especially given that normal care pathways with referral systems in the Spanish hospital system show large proportions of LP and un-tracked origins of referral. These models of care often use point-of-care testing^31^ or reflex testing^32^, helping reduce potential lost to follow-up.

Our findings may be used to identify vulnerable groups (e.g. people who inject drugs and migrants); risk factors, and characteristics (e.g. age) that contribute to LP; track the effectiveness of current interventions in the region; and potentially initiate additional studies and improve timely access to treatment. Given that hospitals serving various and diverse Spanish cities were included in this study, further studies investigating potential barriers to specialist care are warranted.

The main advantages of our study include that it is a multicentre study with a large number of patients included during a two-year period. However, the study has several limitations such as the inconsistency in data quality, including missing data for some variables across centres. Additionally, conflation between late presentation to care and late presentation to treatment should be highlighted. While our study focused on describing late presentation to specialist care because liver specialists are designated to oversee the treatment and care of viral hepatitis patients in Spain, data also show that some patients may have been engaged in care in other specialities before reaching the appropriate specialty for their viral hepatitis care. Additionally, fibrosis stage was reported by each individual participating hospital using different liver staging methods by each. Our data cannot reflect the accuracy of each reported fibrosis stage; hospitals classified their patients’ fibrosis stage according to one of the methods described in “Supplementary material”.

## Conclusions

In conclusion, one in four patients in Spain with chronic viral hepatitis B or C present late to specialist care, of which more than 5% already have irreversible liver damage. Despite universal access to DAA therapy in Spain, late presentation did not decrease from the start of 2018 to the end of 2019. Initiatives to reduce late presentation should specifically target men, foreign-born populations for HBV, Spanish nationals for HCV, and people with past or current drug use, while following the local epidemiology of each autonomous community.

## Supplementary Information

Supplementary Table 1.

## Data Availability

The datasets used and/or analysed during the current study are available from the corresponding author on reasonable request.

## Abbreviations

HBV: Hepatitis B virus
HCV: Hepatitis C virus
CHB: Chronic hepatitis B
CHC: Chronic hepatitis C
WHO: World Health Organization
HCC: Hepatocellular carcinoma
DAAs: Direct-acting antivirals
SVR: Sustained virologic response
LP: Late presentation
ALD: Advanced liver disease
LSLD: Late-stage liver disease
AST: Aspartate aminotransferase
ALT: Alanine transaminase
APRI: Aminotransferase platelet ratio
OR: Odd’s ratio
HIV: Human immunodeficiency virus
