# Supplementary material for: Late presentation of chronic HBV and HCV patients seeking first time specialist care in Spain: a 2-year registry review
Source: Sci Rep. 2021 Dec 17;11:24133. doi: 10.1038/s41598-021-01885-0 (PMC8683418; doi:10.1038/s41598-021-01885-0)
Supplement: Supplementary file 1 — Supplementary Table 1. [file 41598_2021_1885_MOESM1_ESM.docx]

**Supplementary table 1.** Type of serological criteria for fibrosis reported by participating hospitals for HBV and HCV patients.

| **Serological criteria of fibrosis** | **N** | **%** |
| --- | --- | --- |
| APRI Score > 1.5 | 69 | 3.01 |
| FIB-4 > 3.25 | 30 | 1.31 |
| Fibrotest > 0.59 | 3 | 0.13 |
| Fb > 9.5 | 1,166 | 50.92 |
| Missing/ Not reported | 1,021 | 44.58 |
